# Physiological and Transcriptome Analysis of *Polygonatum cyrtonema* Under Continuous Drought Stress

**DOI:** 10.3390/ijms27219670

**Published:** 2026-10-28

**Authors:** Xiurong Xu, Shiming Cheng

**Affiliations:** Zhejiang Academy of Forestry, Hangzhou 310023, China; xuxiurong@zjforestry.ac.cn

**Keywords:** physiological and transcriptome analysis, *P. cyrtonema*, continuous drought stress, WGCNA and PPI analysis

## Abstract

Against the backdrop of global warming and the increasingly uneven distribution of water resources, drought has emerged as a critical abiotic stressor limiting the growth and quality formation of medicinal plants. In this study, *Polygonatum cyrtonema* was subjected to a continuous drought treatment experiment by withholding water for 5, 10, and 20 days, alongside a well-watered control (CK), to preliminarily investigate its physiological responses and transcriptomic alterations under water-deficit conditions. Physiological measurements revealed that prolonged drought stress significantly increased the concentrations of malondialdehyde (MDA) and hydrogen peroxide (H_2_O_2_) in leaves, accompanied by elevated in superoxide dismutase (SOD) and peroxidase (POD) activities, as well as increased chlorophyll content. RNA-seq analysis identified a substantial number of differentially expressed genes (DEGs), with 11,835, 12,110, and 11,416 DEGs in the D5, D10, and D20 vs. CK comparisons, respectively, including 6133 shared core DEGs. KEGG enrichment analysis indicated that these DEGs were primarily involved in starch and sucrose metabolism, phenylpropanoid biosynthesis, and plant hormone signal transduction. Weighted gene co-expression network analysis (WGCNA) identified a specific gene module (black module) containing 1681 genes that was highly positively correlated with drought duration (r = 0.95). Protein–protein interaction (PPI) network analysis of this module highlighted several candidate hub genes, including WRKY33 (BMK_Unigene_251623), EDA39 (BMK_Unigene_034545), OHP (BMK_Unigene_221621), and RD20 (BMK_Unigene_149641). Furthermore, genes within the black module were significantly enriched in metabolic pathways such as anthocyanin, phenylpropanoid, and isoflavonoid biosynthesis, as well as phenylalanine, tyrosine, tryptophan, arginine, and proline metabolism. RT-qPCR validation confirmed the expression patterns of these candidate genes, supporting the reliability of the transcriptomic data. Collectively, these findings provide transcriptomic insights into the continuous drought response in *P. cyrtonema*, and identify candidate genes and pathways for future functional validation and stress-resilience improvement in this species.

## 1. Introduction

Under the dual pressures of global warming and uneven water resource distribution, drought has emerged as a critical abiotic stressor limiting the growth and quality formation of medicinal plants [1,2]. *P. cyrtonema*, a perennial herb belonging to the genus *Polygonatum* in the family Liliaceae, is rich in bioactive compounds such as polysaccharides, saponins, and flavonoids [3,4,5,6,7]. As a well-known traditional Chinese medicine, *P. cyrtonema* possesses various pharmacological effects, including hypoglycemic activity, lipid regulation, and immune enhancement, with a long history of clinical application [5,6,7]. Despite the expanding scale of artificial cultivation driven by increasing market demand, the long growth cycle and high environmental sensitivity of *P. cyrtonema* remain significant challenges. In drought-prone regions, water deficiency substantially impairs its growth, development, secondary metabolite biosynthesis, and final yield, thereby restricting the sustainable development of the industry.

Drought stress, resulting from the scarcity of available water, leads to an imbalance between water supply and demand, serving as a primary environmental factor limiting plant productivity [8]. In the early stages of drought, plants primarily mitigate water loss by inducing stomatal closure, a process predominantly regulated by abscisic acid (ABA) through a cascade of signaling components within guard cells [9]. Additionally, ABA signaling promotes primary root elongation to access deeper soil moisture [10]. Prolonged drought activates antioxidant enzyme systems and induces the accumulation of osmotic adjustments, such as proline, betaine, and soluble sugars [11,12]. To cope with drought, plants coordinate gene expression via complex molecular regulatory networks to achieve adaptive physiological and metabolic shifts [13,14,15]. Transcriptomics has become an essential tool for elucidating stress-response mechanisms, enabling the systematic revelation of genome-wide expression dynamics and the identification of key functional genes and signaling pathways [16]. High-throughput sequencing has been widely applied to study stress biology in medicinal plants, providing robust support for identifying stress-resistance genes and deciphering secondary metabolic regulation. While drought-responsive transcriptomes have been reported for several medicinal species, such as *Glycyrrhiza uralensis* [1] and *Sophora alopecuroides* [17], research on *Polygonatum* species has primarily focused on rhizome polysaccharide accumulation under normal conditions [18]. How drought stress specifically reprograms the transcriptome linking osmotic adjustment with secondary metabolite (e.g., flavonoids and saponins) biosynthesis in *P. cyrtonema* remains largely unknown. In particular, systematic analyses at the whole-transcriptome level are lacking, and existing studies have not dynamically tracked the temporal regulatory networks from osmotic adjustment to secondary metabolism under continuous drought, leaving the key regulatory nodes (e.g., specific transcription factors) unidentified.

Investigating the transcriptome of *P. cyrtonema* under drought stress will not only reveal the molecular basis of gene expression reprogramming but also identify differentially expressed genes (DEGs) involved in crucial pathways such as osmotic adjustment, antioxidant defense, hormone signaling (e.g., the ABA pathway), and secondary metabolism (e.g., anthocyanin, flavonoid, and isoflavonoid biosynthesis) [19,20,21]. Furthermore, previous studies have demonstrated that transcription factors such as WRKY, NAC, and MYB play pivotal roles in regulating plant drought tolerance [22,23,24]. Screening these candidate regulators and functional genes through RNA-seq will provide a theoretical foundation for understanding the molecular mechanisms of drought resistance in *P. cyrtonema*. These findings will offer valuable genetic resources and scientific support for breeding stress-resistant varieties, optimizing standardized cultivation techniques, and regulating medicinal quality.

We hypothesized that continuous drought stress triggers coordinated transcriptional reprogramming in *P. cyrtonema*, in which specific transcription factors (e.g., WRKY) mediate the crosstalk between antioxidant defense and secondary metabolite biosynthesis. To test this hypothesis, we conducted a preliminary investigation of physiological and transcriptomic changes in *P. cyrtonema* under a continuous drought treatment using RNA-seq technology. The objectives were to characterize the gene expression profiles, identify core responsive genes, construct regulatory networks, and screen candidate genes and pathways as a reference for future functional studies. The results of this research will enrich the theoretical framework of stress biology in medicinal plants and provide a scientific basis for the future genetic enhancement of drought tolerance in *P. cyrtonema*.

## 2. Results

### 2.1. Changes in Physiological and Biochemical Indicators of P. cyrtonema Under Continuous Drought Stress

**Figure 1:**
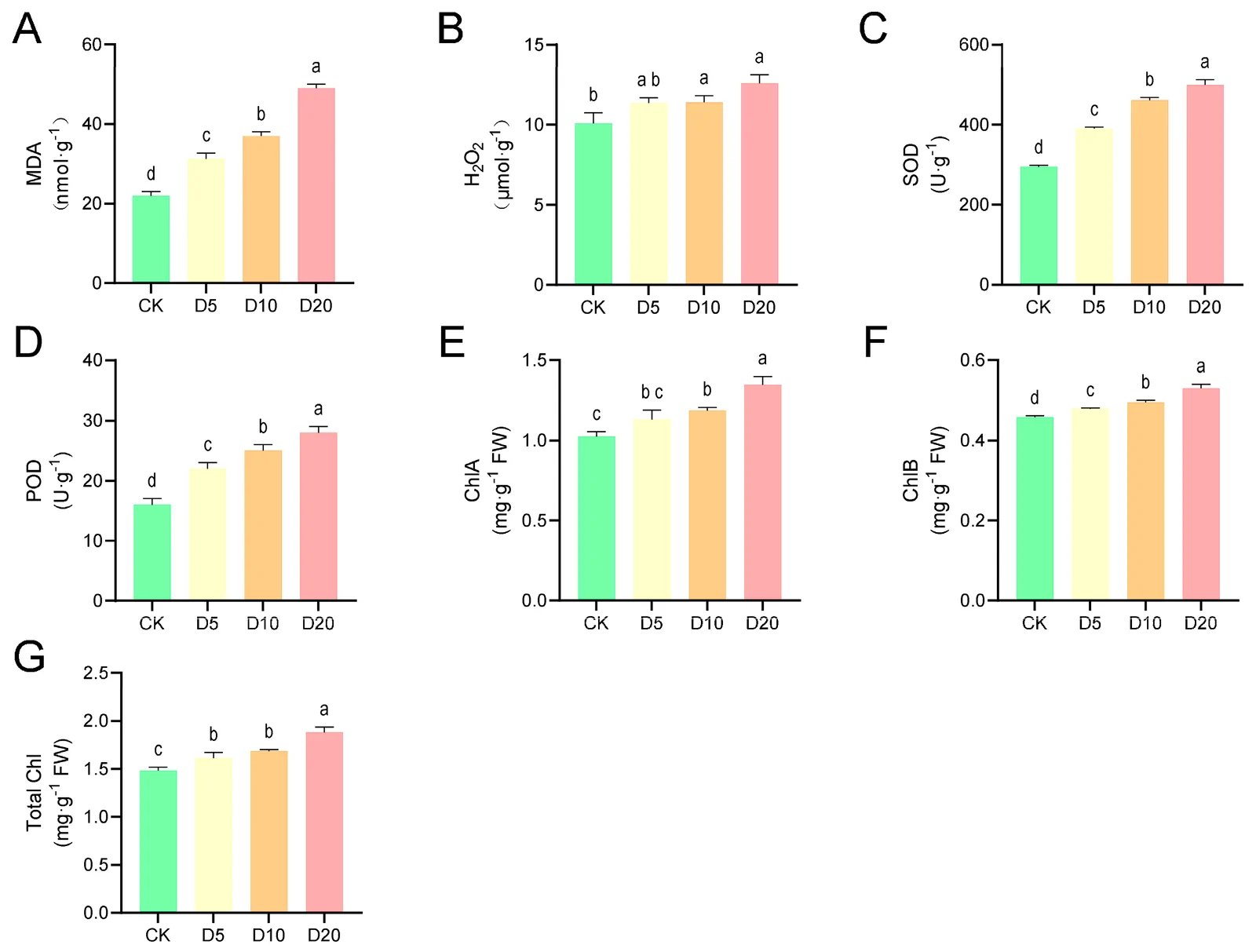
The changes in the physiological indicators of multi-flowered *P. cyrtonema* under drought stress. Different letters above the column indicate significant differences. D5, D10, D20 respectively represent the number of days of continuous drought. (**A**) Malondialdehyde content, (**B**) hydrogen peroxide content, (**C**) superoxide dismutase activity, (**D**) peroxidase activity, (**E**) chlorophyll A content, (**F**) chlorophyll B content, (**G**) total chlorophyll content.

Under continuous water deprivation, the levels of malondialdehyde (MDA) content in the leaves of *P. cyrtonema* increased significantly from 22 μmol/g (CK) to 49 μmol/g (D20), and hydrogen peroxide (H_2_O_2_) increased from 10.1 μmol/g to 12.6 μmol/g (Figure 1A,B). Similarly, the activities of superoxide dismutase (SOD) and peroxidase (POD) exhibited a significant upward trend over the stress period, increasing from 294.67 U/g to 499.67 U/g (Figure 1C,D) the levels of malondialdehyde (MDA) and hydrogen peroxide (H_2_O_2_) in the leaves of *P. cyrtonema* increased significantly as the drought duration extended (Figure 1A,B). Similarly, the activities of superoxide dismutase (SOD) and peroxidase (POD) exhibited a significant upward trend over the stress period (Figure 1C,D). Furthermore, the leaf chlorophyll content also increased progressively with prolonged drought exposure (Figure 1E–G).

### 2.2. Transcriptome Data Quality Control and Inter-Sample Correlation Analysis

**Figure 2:**
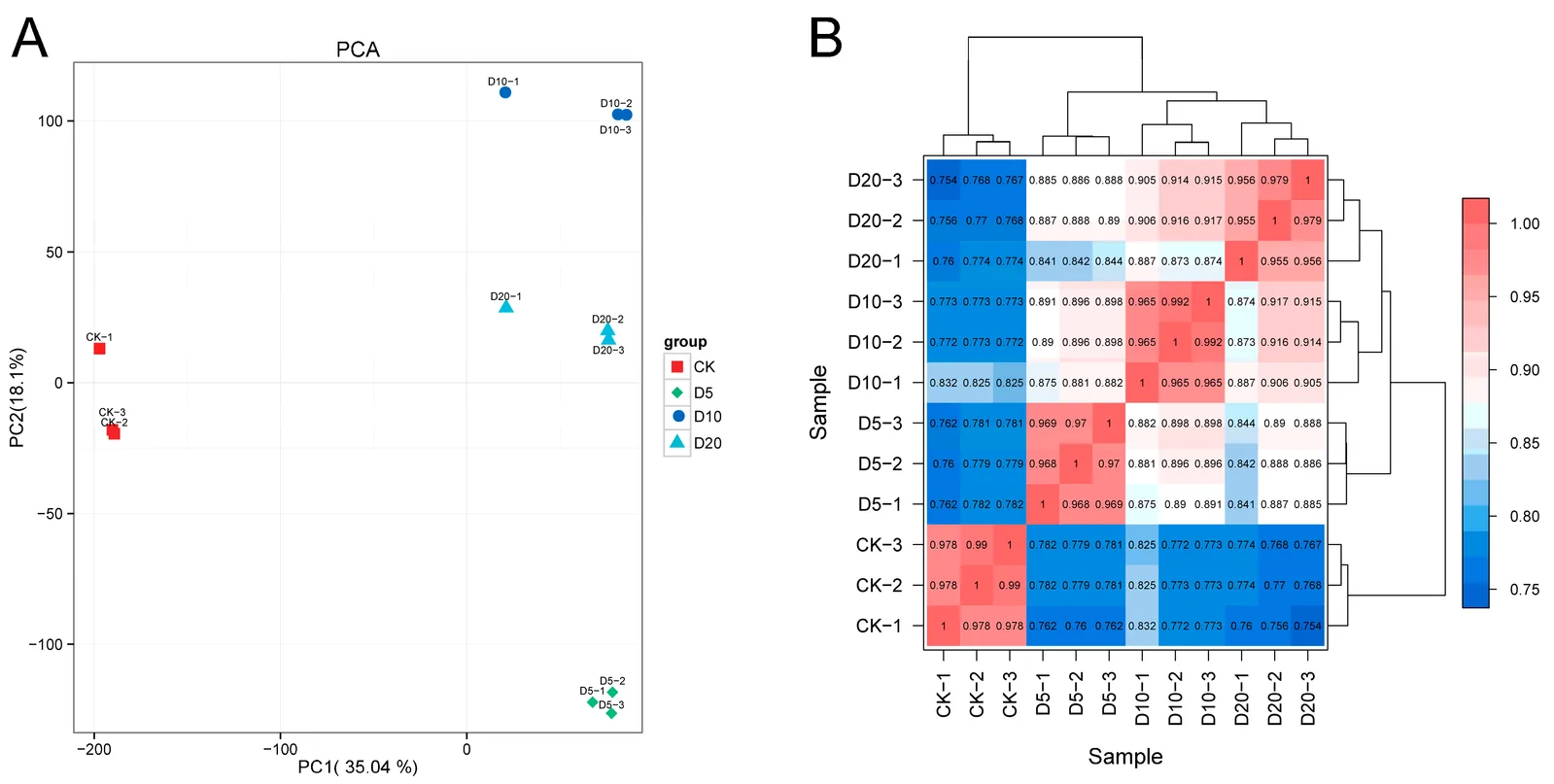
(**A**) Principal component analysis (PCA) and (**B**) inter-sample correlation analysis among groups based on the FPKM values of all genes in the *P. cyrtonema* transcriptome under drought stress.

RNA sequencing generated a total of 125.1 GB of clean data. The GC content across samples ranged from 45.72% to 47.81%, and the Q30 base percentage exceeded 90% for all samples, confirming the high reliability of the sequencing data (Supplementary Table S2). Based on the FPKM values of all 53,366 identified genes (after filtering genes with FPKM ≤ 1 in all samples, 38,214 genes were retained for downstream analysis), principal component analysis (PCA) and Pearson correlation analyses were performed on the 12 samples. The PCA plot demonstrated a clear separation among the CK, D5, D10, and D20 treatment groups, indicating that drought stress significantly altered global gene expression in *P. cyrtonema* (Figure 2A). The first two principal components (PC1 and PC2) cumulatively explained 53.14% of the overall variance, suggesting substantial biological variation or the influence of uncontrolled micro-environmental factors. Nevertheless, the clear separation among the CK, D5, D10, and D20 groups along PC1 indicates that drought duration is a dominant driving force of transcriptomic changes. To mitigate the impact of high intra-group variation, we applied stringent thresholds (FDR < 0.01 and FC ≥ 2) for DEG identification and utilized WGCNA to identify co-expression modules robust to individual gene noise.

D5, D10, D20 respectively represent the number of days of continuous drought.

### 2.3. Identification of Differentially Expressed Genes and KEGG Pathway Enrichment Analysis

**Figure 3:**
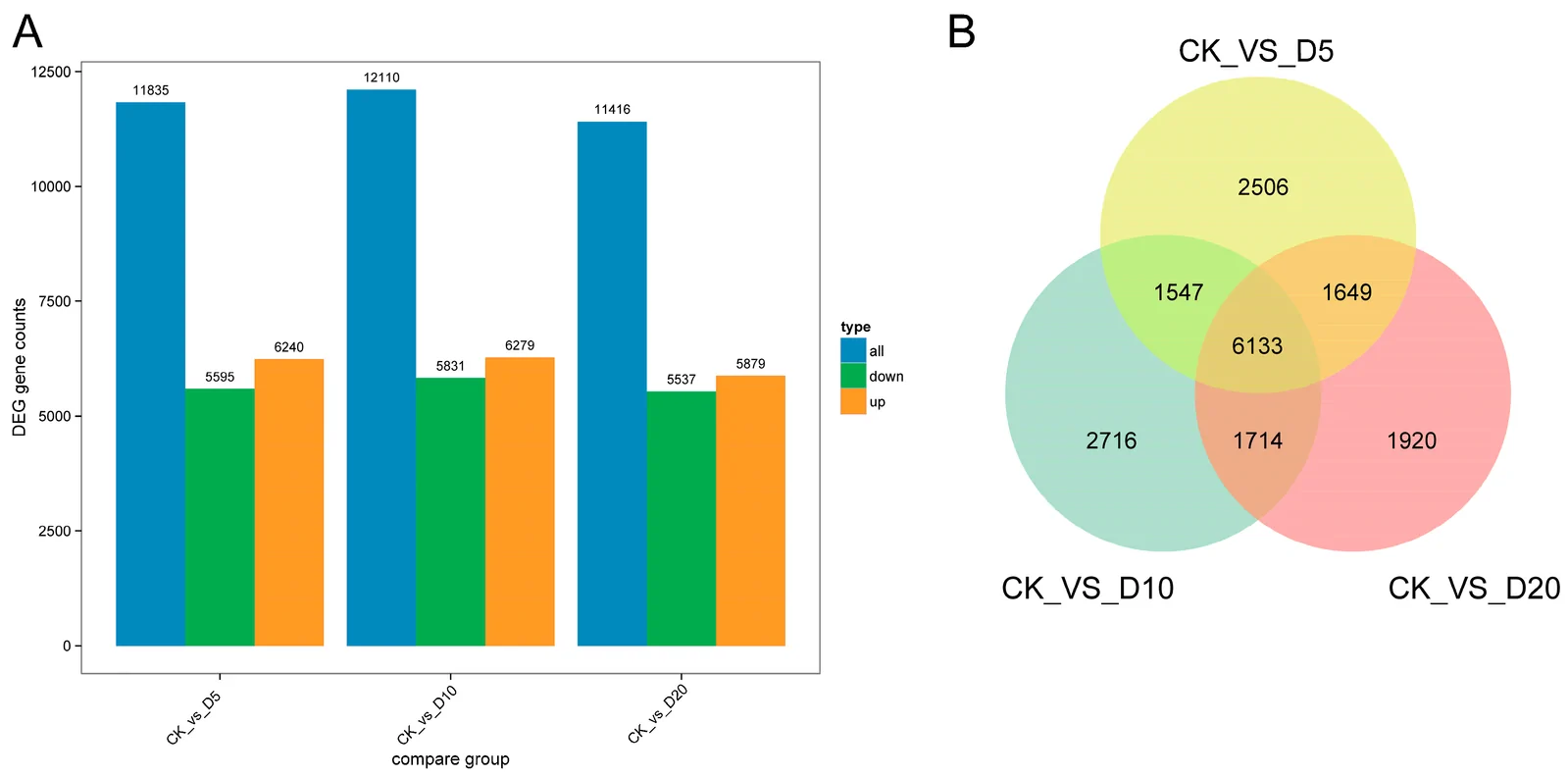
(**A**) Statistical analysis of differentially expressed genes (DEGs) in the transcriptome of *Polygonatum cyrtonema* under drought stress, and (**B**) Venn diagram showing the overlap of DEGs among different treatments.

**Figure 4:**
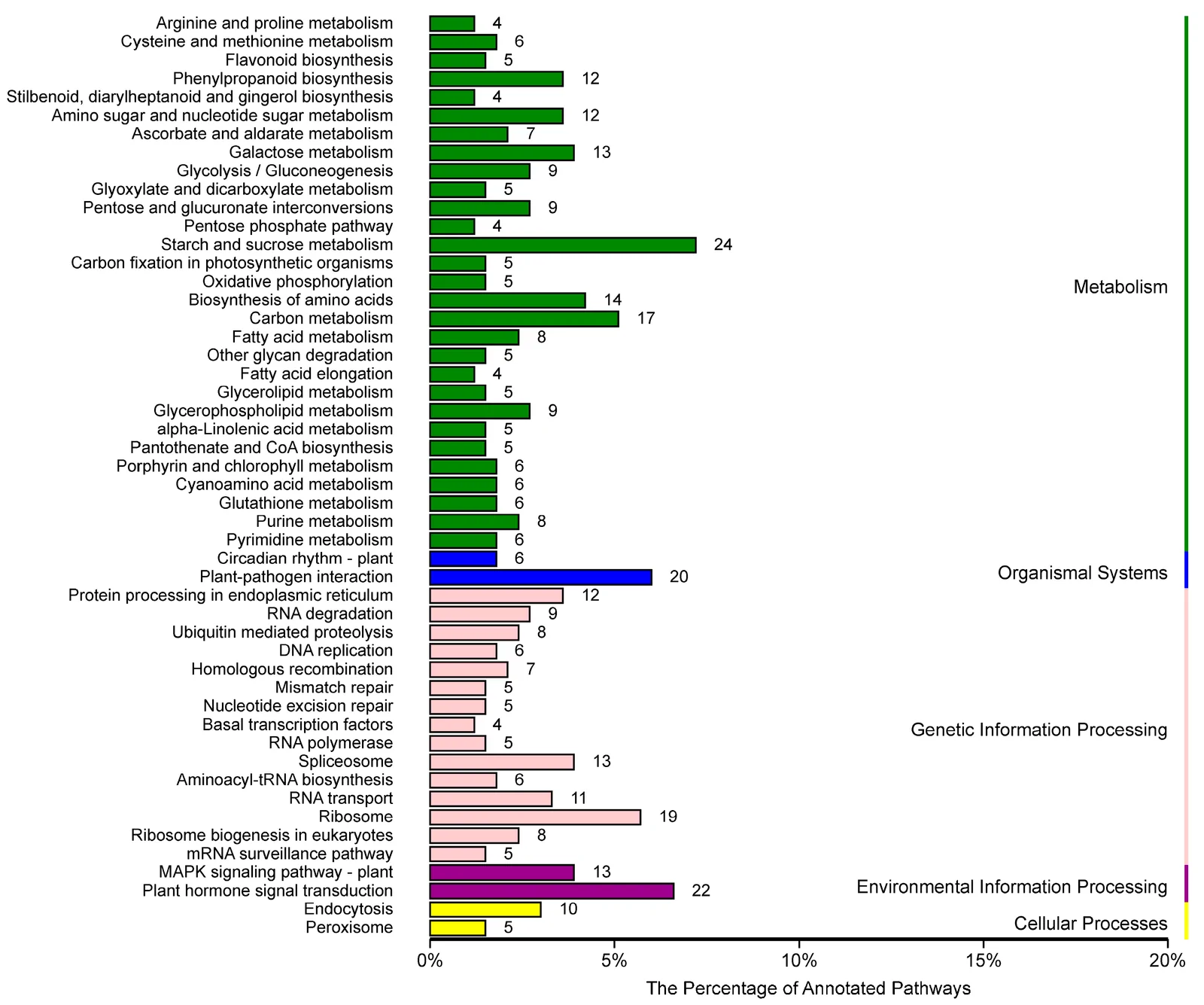
KEGG enrichment analysis diagram of differentially expressed genes in *P. cyrtonema* under drought stress.

Transcriptomic analysis identified substantial transcriptomic reprogramming in response to continuous water deprivation. Compared to the control (CK), 11,835 differentially expressed genes (DEGs) were identified in the D5 treatment (6240 upregulated and 5595 downregulated). In the D10 treatment, 12,110 DEGs were detected (6279 upregulated and 5831 downregulated), while the D20 treatment yielded 11,416 DEGs (5879 upregulated and 5537 downregulated) (Figure 3A). A Venn diagram analysis revealed a core set of 6133 DEGs shared across all drought durations (Figure 3B). KEGG pathway annotation of these 6133 shared DEGs demonstrated significant enrichment in pathways associated with metabolism, organismal systems, genetic information processing, environmental information processing, and cellular processes. Notably, the pathways containing the highest numbers of DEGs included starch and sucrose metabolism, phenylpropanoid biosynthesis, and plant hormone signal transduction (Figure 4). The proportion of DEGs relative to the total unigenes (approximately 22–23%) is higher than typically observed in drought studies, which maybe attributable to the compounded stress of high background temperature (35–40 °C) and severe drought, which extensively reprogramming gene expression. Additionally, the relatively low PCA variance explained suggests notable biological variation, which may inflate DEG numbers. We applied a stringent FDR (<0.01) to control false discoveries; however, future studies under controlled temperature conditions are needed to refine these expression profiles.

### 2.4. WGCNA and PPI Analysis

**Figure 5:**
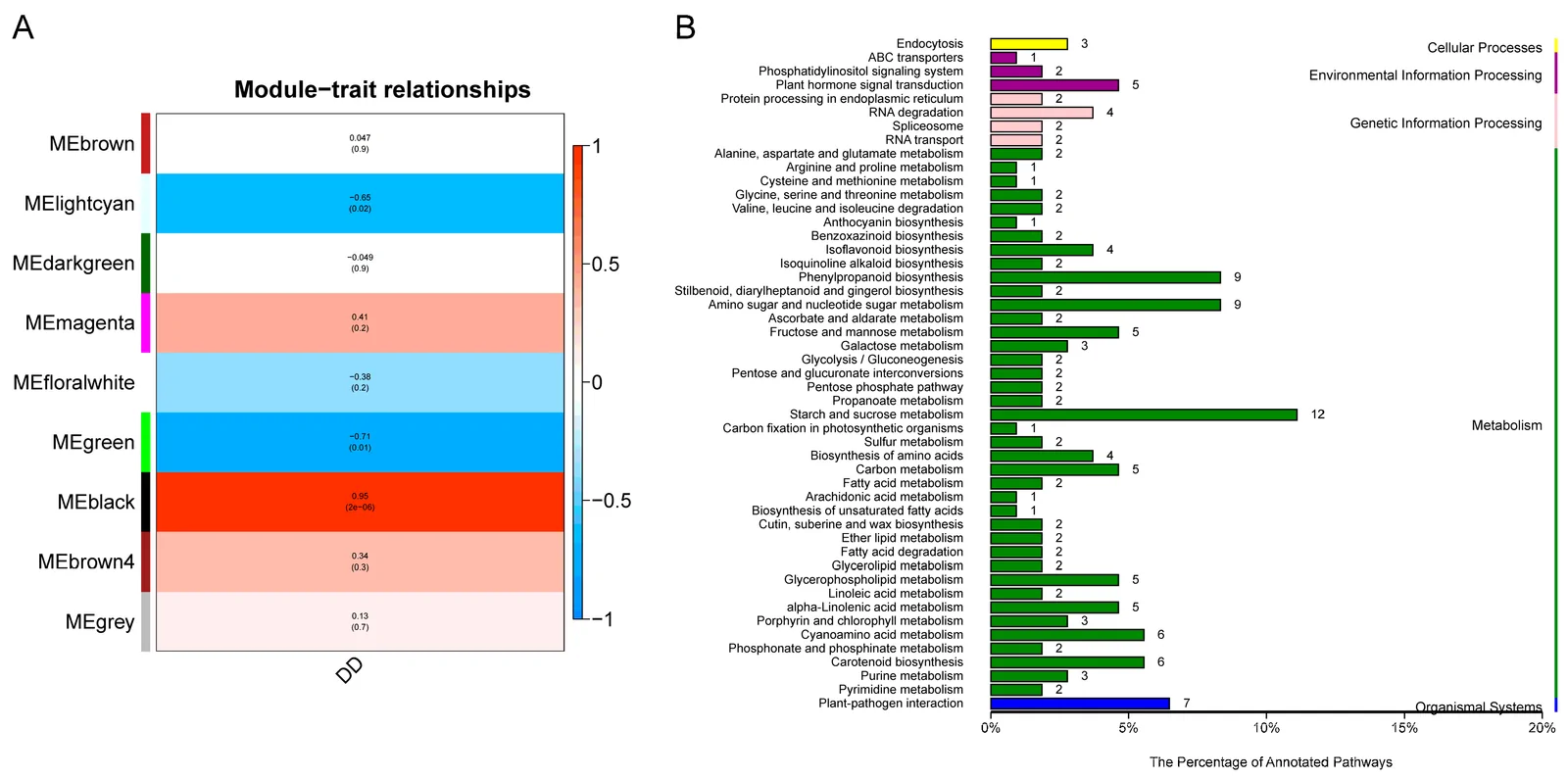
(**A**) Correlation analysis between WGCNA co-expression modules and the duration of drought stress (days). (**B**) KEGG pathway enrichment analysis of genes in the black module. DD indicates the degree of drought.

Weighted gene co-expression network analysis (WGCNA) was performed on all identified DEGs, clustering them into nine distinct co-expression modules (Supplementary Figure S1). Module-trait correlation analysis revealed that the module most highly correlated with drought duration was labeled as ‘MEblack’ (r = 0.95, *p* = 0.001) (Figure 5A). Hereafter, we refer to this as the drought-associated black module. Other modules also showed notable correlations with specific physiological traits: the “turquoise” module was significantly correlated with MDA content (r = 0.82), while the “blue” module showed positive correlation with SOD activity (r = 0.78), suggesting their respective roles in oxidative stress response regulation. KEGG functional annotation of the genes within each module showed that most were enriched in pathways related to metabolism, organismal systems, genetic information processing, environmental information processing, and cellular processes. Among these, starch and sucrose metabolism, phenylpropanoid biosynthesis, and plant hormone signal transduction contained the highest number of DEGs (Figure 5B). To predict protein–protein interactions, the protein sequences of the 1681 members of the black module were uploaded to the STRING database (Supplementary Figure S2A), resulting in a network with 104 nodes. Functional annotation identified *RD20* (BMK_Unigene_149641) as a candidate gene closely associated with drought stress, suggesting the potential biological relevance of the black module to the drought response, pending further experimental validation (Supplementary Table S3). Subsequent topological analysis using Cytoscape (Supplementary Figure S2B) revealed that *WRKY33* (BMK_Unigene_251623), *EDA39* (BMK_Unigene_034545), *OHP* (BMK_Unigene_221621), and *RD20* (BMK_Unigene_149641) exhibited high degree values, indicating their central roles as candidate hub proteins within the interaction network (Supplementary Table S3). However, it should be noted that not all biological functions are evolutionarily conserved, which may result in the omission or misinference of PPI relationships specific to *P. cyrtonema*. These candidate genes require further functional validation through gene knockout, overexpression, or protein interaction experiments before their regulatory roles in *P. cyrtonema* drought response can be confirmed.

### 2.5. Validation of Transcriptome Data via RT-qPCR

**Figure 6:**
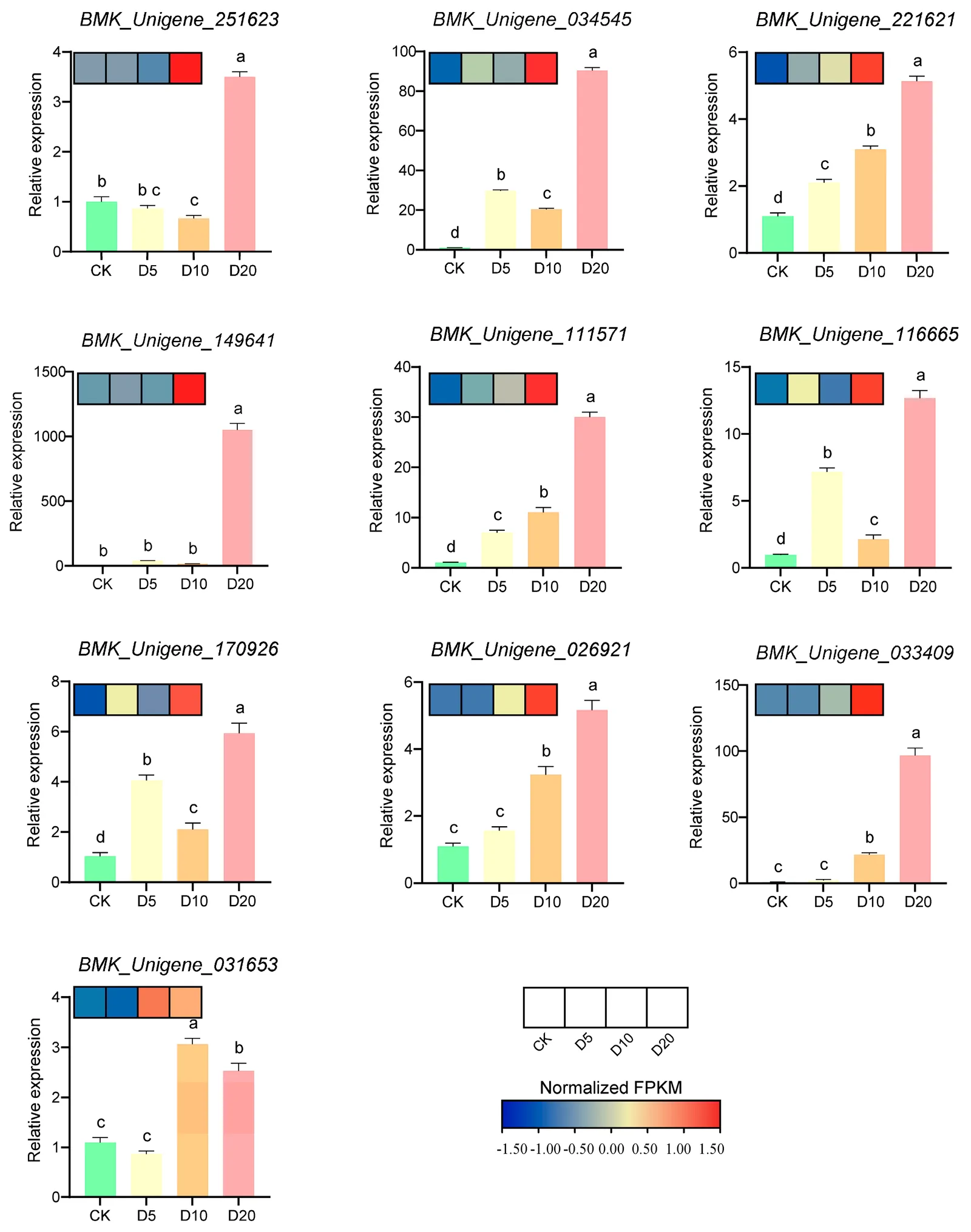
RT-qPCR verified the relative expression levels of key drought-related genes. Different letters above the bars indicate significant differences.

To experimentally validate the reliability of the RNA-seq expression profiles, 10 candidate drought-responsive genes were selected for RT-qPCR analysis (Figure 6). The expression patterns of all 10 selected genes showed high consistency between RT-qPCR and RNA-seq data, with a Pearson correlation coefficient (R^2^) of 0.85 (*p* < 0.01), confirming the reliability of the transcriptomic profiling. The *P. cyrtonema* ubiquitin gene was used as an endogenous reference, as its expression stability across all drought treatments was verified by our RNA-seq data (coefficient of variation in FPKM values < 5% across all samples). These included four candidate hub genes from the PPI network (*BMK_Unigene_251623*, *BMK_Unigene_034545*, *BMK_Unigene_221621*, and *BMK_Unigene_149641*) and six genes from the black module representing critical metabolic pathways: anthocyanin biosynthesis (*BMK_Unigene_111571*); phenylalanine, tyrosine, and tryptophan biosynthesis (*BMK_Unigene_116665*); arginine and proline metabolism (*BMK_Unigene_170926*); starch and sucrose metabolism (*BMK_Unigene_026921*); phenylpropanoid biosynthesis (*BMK_Unigene_033409*); and isoflavonoid biosynthesis (*BMK_Unigene_031653*). The high consistency between the RT-qPCR relative expression levels and the transcriptomic FPKM values confirmed the accuracy of the sequencing data. Furthermore, the significant differential expression of these genes under water deficit highlights their critical roles in the drought stress response of *P. cyrtonema*.

## 3. Discussion

*P. cyrtonema* is a vital medicinal plant whose yield has been significantly compromised in recent years by global warming and the increasingly uneven distribution of water resources. To address this, the present study conducted a preliminary physiological and transcriptomic investigation of *P. cyrtonema* subjected to a continuous drought-duration treatment. By screening candidate drought-responsive genes and regulatory pathways, this research provides reference information for future functional studies and cultivation strategies aimed at improving stress resilience in *P. cyrtonema*.

Exposure to abiotic stress often triggers the overproduction of reactive oxygen species (ROS), including free radicals that oxidize membrane unsaturated fatty acids, leading to membrane damage and the accumulation of malondialdehyde (MDA). ROS also include non-radical oxidants such as hydrogen peroxide (H_2_O_2_), which can be converted into highly toxic hydroxyl radicals [25]. Our findings show that both MDA and H_2_O_2_ levels in *P. cyrtonema* increased progressively with drought duration. These results align with observations in other species, such as *Brassica napus* [26], *Camellia sinensis* [27], and *Medicago sativa* [28], confirming that *P. cyrtonema* experienced significant oxidative stress during the 20-day water deprivation period. The concomitant increase in REC further supports the conclusion that membrane integrity was progressively compromised under prolonged water deficit. To mitigate ROS-induced damage, plants employ antioxidant defense systems, primarily involving enzymes such as superoxide dismutase (SOD) and peroxidase (POD) [29,30]. In this study, SOD and POD activities increased alongside drought duration, indicating an active enzymatic defense response in *P. cyrtonema*. This continuous upregulation of antioxidant enzymes over 20 days suggests that the drought conditions applied in this study, although progressively intensifying, may not have exceeded the threshold at which the antioxidant defense system collapses in *P. cyrtonema*. It is possible that this species maintains ROS homeostasis through enhanced enzymatic activity over a relatively extended drought period. Nevertheless, whether this reflects a genuinely superior antioxidant capacity or simply the response to a sub-lethal drought duration remains to be determined in future studies using more severe drought treatments.

Interestingly, while chlorophyll content typically declines under drought, we observed an increase. While the ‘dehydration-induced concentration effect’ due to leaf dehydration provides a partial explanation, the shade-adapted nature of *P. cyrtonema* may offer an additional biological rationale. Shade-tolerant species often prioritize maintaining light-harvesting capacity under stress to compensate for reduced photosynthetic efficiency. This may represent an adaptive response to sustain energy supply, although this hypothesis requires validation using chlorophyll fluorescence and dry-weight-based quantification.” We propose that this unexpected result may be partially explained by a “concentration effect” caused by progressive leaf dehydration: as leaf water content decreases (evidenced by declining RWC), chlorophyll mass per unit fresh weight may increase even if total chlorophyll synthesis remains unchanged or declines slightly [31,32]. In addition, *P. cyrtonema* is a shade-adapted species that may sustain or even enhance its light-harvesting capacity under moderate stress by maintaining chlorophyll levels. However, this interpretation requires further validation using parameters such as chlorophyll fluorescence (Fv/Fm), photosynthetic gas exchange rates, and dry-weight-based chlorophyll quantification. We therefore present this finding cautiously and acknowledge that the underlying mechanism remains unclear.

At the molecular level, transcriptome analysis revealed that DEGs were significantly enriched in starch and sucrose metabolism, phenylpropanoid biosynthesis, and plant hormone signal transduction. Starch and sucrose metabolism, the most enriched pathway, is directly linked to the accumulation of soluble sugars, which serve as crucial osmoprotectants [33]. In *P. cyrtonema*, soluble sugars may also contribute directly to the biosynthesis of polysaccharides, which are the primary bioactive compounds of this medicinal plant. Therefore, the upregulation of genes in this pathway under drought may simultaneously reflect osmotic adjustment and secondary metabolite accumulation, suggesting a potential dual function. Increased soluble sugar content has been shown to enhance drought tolerance in apples [34], tomatoes [35], and mulberries [36]. Similarly, the enrichment of phenylpropanoid biosynthesis and hormone signaling pathways is consistent with findings in tobacco [37], rice [38], and coconut [39], where these pathways regulate physiological adaptation to water deficit, often mediated by abscisic acid (ABA).The phenylpropanoid pathway is also directly linked to the biosynthesis of flavonoids, which are important antioxidant compounds in *P. cyrtonema*, suggesting that drought stress may simultaneously promote both stress defense and medicinal quality-related secondary metabolite accumulation. While previous studies in medicinal plants primarily focused on conserved ABA signaling, our identification of the OHP and EDA39 nodes within the drought-correlated black module suggests a potential integration of light-stress signaling with drought responses in *P. cyrtonema*. This extends the current understanding of drought-responsive networks in shade-tolerant medicinal monocots beyond the canonical ABA/ROS pathways.

To identify candidate regulatory components, we utilized WGCNA and PPI network analyses, identifying several high-degree candidate hub genes requiring further functional validation. Among these, *BMK_Unigene_251623* encodes a WRKY33 transcription factor. WRKY factors are well-documented regulators of abiotic stress responses [40]; for instance, *LcWRKY14* enhances drought resistance in *Litsea cubeba* by promoting tannin biosynthesis [41], while *TaWRKY76* in wheat regulates soluble sugars and proline levels [42]. In *P. cyrtonema*, WRKY33 is proposed as a candidate transcriptional regulator of drought-responsive gene expression, although its specific targets and regulatory mechanisms in this species have not yet been experimentally verified. *RD20* (*BMK_Unigene_149641*), a member of the caleosin family, was also identified as a candidate key node. *RD20* is known to act as a peroxygenase involved in oxylipin metabolism during stress, and its expression is highly inducible by drought in leaves and flowers. Furthermore, *EDA39* (*BMK_Unigene_034545*), an IQ domain-containing protein, likely modulates stomatal movement, while *OHP* (*BMK_Unigene_221621*), a light-harvesting complex-like protein, may protect photosynthetic machinery from photo-oxidation under drought conditions [43,44,45]. These candidate genes are proposed based on network topology and functional annotation; their roles in *P. cyrtonema* drought response remain to be confirmed through gene-specific functional experiments such as overexpression or RNA interference. Given the large evolutionary distance between *P. cyrtonema* (monocot) and *A. thaliana* (dicot), some inferred PPIs may not be biologically valid. To mitigate this cross-species inference limitation, we strictly filtered candidate hub genes by requiring them to possess both high degree values in the PPI network and high intramodular connectivity (kME) in the WGCNA black module, alongside functional annotation supporting drought responsiveness.

In addition to these candidate hub genes, we focused on DEGs within the “black” module associated with the biosynthesis of anthocyanins, isoflavonoids, and phenylpropanoids, as well as the metabolism of amino acids (proline, phenylalanine, tyrosine, and tryptophan). These pathways produce metabolites essential for enhancing drought resistance [17,46,47,48,49,50,51]. RT-qPCR validation confirmed that genes involved in these pathways were significantly upregulated after 20 days of drought. Collectively, these results suggest that *P. cyrtonema* responds to continuous water deficit through a multifaceted strategy that tentatively involves osmotic adjustment, antioxidant defense, and activation of specialized secondary metabolic pathways. These findings provide a preliminary transcriptomic framework for understanding the drought response of this species. Transcriptomic analysis identified a massive number of DEGs (approximately 22–23% of all unigenes), which is higher than typically observed in drought studies. This is likely attributable to the severe drought, which extensively reprogram gene expression. Additionally, the relatively low PCA variance explained suggests notable biological variation, which may inflate DEG numbers. We applied a stringent FDR (<0.01) to control false discoveries; however, future studies under controlled temperature conditions are needed to refine these expression profiles.

Collectively, we propose a preliminary regulatory network for drought response in *P. cyrtonema*: Prolonged drought triggers ROS accumulation and membrane damage, activating antioxidant enzymes (SOD, POD). Concurrently, drought-responsive TFs (e.g., WRKY33) are induced, which orchestrate the reprogramming of metabolic flux towards osmolyte accumulation (sucrose, proline) and secondary metabolite biosynthesis (phenylpropanoids, flavonoids), thereby integrating stress defense with potential medicinal quality modulation. Although direct functional validation is lacking, our co-expression network suggests a putative regulatory model where WRKY33 may act as a central transcriptional activator coordinating the expression of phenylpropanoid and isoflavonoid biosynthetic genes under drought stress. This model provides a testable hypothesis for future functional genomics studies in *P. cyrtonema*.

## 4. Materials and Methods

### 4.1. Plant Materials and Drought Treatment

Three-year-old *P. cyrtonema* ‘Lijing No. 1’ seedlings, cultivated in a greenhouse at the Zhejiang Academy of Forestry, were used as the experimental material. The experiment commenced on 15 July 2025. A total of 120 plants with uniform growth were selected and divided into a control group (CK) and three drought treatment groups, with 30 plants per group. The greenhouse environment was maintained at 20–30 °C with a 12 h light/12 h dark photoperiod. Plants were cultivated in plastic pots (20 cm diameter × 25 cm height) filled with a substrate consisting of 20% peat, 10% perlite, 70% local loam soil. All plants were subjected to the same temperature regime; thus, observed physiological and transcriptomic differences were primarily attributed to water availability. Nevertheless, we acknowledge that the relatively high background temperature may have interacted with drought stress, and this is considered as a limitation of the present study. During the experimental period, soil water content (SWC) was monitored using a portable soil moisture meter (TSC-1, Shijiazhuang, China) and in the drought treatment groups, SWC declined progressively from 60% (field capacity) at day 0 to approximately 35%, 20%, and 10% at days 5, 10, and 20, respectively, confirming the establishment of a continuous drought-duration gradient. The CK group was maintained at 60% SWC throughout the experiment by regular irrigation every three days. All groups were watered every three days and subjected to routine management.

Samples were collected on day 0 (CK), day 5 (D5), day 10 (D10), and day 20 (D20) of the drought treatment. For each sampling, fully expanded leaves at the same developmental stage, specifically the third to fifth leaves from the shoot apex, were collected at the same time of day (09:00–10:00 a.m.) from each plant. One leaf was harvested from each of the 30 plants per group, and ten leaves were pooled to form a single biological sample, yielding three biological replicates per group. All samples were immediately frozen in liquid nitrogen and stored at −80 °C until further analysis. This sample size and pooling strategy were based on previous transcriptomic studies in medicinal plants under abiotic stress, which demonstrated robust detection of differentially expressed genes with similar replicates.

### 4.2. Determination of Physiological and Biochemical Indices

The concentrations of malondialdehyde (MDA) and hydrogen peroxide (H_2_O_2_), the activities of superoxide dismutase (SOD) and peroxidase (POD), and total chlorophyll content were determined by “Wuhan ProNets Testing Technology Co., Ltd. (Wuhan, China). Specifically, MDA content was measured using the thiobarbituric acid (TBA) method [25], while H_2_O_2_ levels were quantified via the titanium sulfate method [52]. SOD activity was determined using the nitroblue tetrazolium (NBT) reduction assay [52], and POD activity was measured following the H_2_O_2_-dependent oxidation method [52]. Chlorophyll content was determined using the ethanol extraction method [53]. In addition, leaf relative water content (RWC) was measured using the fresh weight–turgid weight–dry weight method. Proline content was determined using the sulfosalicylic acid method. Soluble sugar content was measured using the anthrone colorimetric method. Relative electrical conductivity (REC) was determined using a conductivity meter to assess cell membrane integrity.

### 4.3. RNA Sequencing and Differential Gene Expression Analysis

RNA sequencing was performed on the Illumina NovaSeq 6000 platform by Biomarker Technologies (Qingdao, China). Raw sequencing data were filtered to obtain high-quality clean reads, which were subsequently subjected to de novo assembly using Trinity software (v2.11.0) with default parameters (min_kmer_cov: 2, KMER_SIZE: 25). Assembly quality was evaluated using TransRate (v1.0.3) and BUSCO (v5.2.2) against the embryophyta_odb10 dataset to assess completeness. Redundant sequences were removed using CD-HIT-EST (v4.8.1) at a 95% identity threshold to yield the final set of unigenes. For annotation, unigenes were aligned against the NR, Swiss-Prot, and KEGG databases using BlastX-2.13.0 with an E-value threshold of 1 × 10^−5^.

Gene expression levels were quantified using Feature Counts (http://subread.sourceforge.net/, accessed on 15 January 2026), and fragments per kilobase of transcript per million mapped reads (FPKM) values were calculated to normalize expression based on transcript length. To reduce noise from low-abundance transcripts, only genes with FPKM > 1 in at least one sample were retained for downstream analysis. To reduce noise from low-abundance transcripts, only genes with FPKM > 1 in at least one sample were retained for downstream analysis, A soft-thresholding power (β) of 18 was selected to achieve a scale-free topology fit index (R^2^) of 0.85. Differential expression analysis was performed based on the read counts of each sample using the DESeq2 package (v1.4.5). The thresholds for identifying differentially expressed genes (DEGs) were set as a fold change (FC) ≥ 2 and a false discovery rate (FDR) < 0.01. The fold change represents the ratio of expression levels between two samples, while the FDR is the adjusted *p*-value used to determine the statistical significance of the differential expression. Subsequent functional data mining of the DEGs was conducted using the BMKCloud platform, which included the identification of shared DEGs via Venn diagrams, as well as KEGG annotation and pathway enrichment analyses.

### 4.4. Weighted Gene Co-Expression Network Analysis (WGCNA) and Protein-Protein Interaction (PPI) Network Analysis

WGCNA was performed on all DEGs using the WGCNA R package, with the following parameters: FPKM > 1, minimum module size of 30, and a merge cut height of 0.25 [54]. For WGCNA, the soft-thresholding power was set to 18 based on the scale-free topology model fit (R^2^ > 0.85) to achieve approximate scale-free topology. All genes within the identified “black” module were uploaded to the STRING database (https://version-11-0.string-db.org/) for analysis, using *Arabidopsis thaliana* as the reference species to construct the interaction network. The network was further processed and visualized using Cytoscape v3.10.0, where protein degree values were calculated to identify candidate hub genes. The final interaction networks were generated using both STRING and Cytoscape. Hub genes were defined as genes with absolute module membership (MM) > 0.8 and gene significance (GS) > 0.6. The selection of WRKY33, RD20, OHP, and EDA39 was justified by their high intramodular connectivity (kME > 0.85) and significant correlation with drought duration (*p* < 0.01), meeting the stringent hub gene criteria. Protein–protein interaction networks were constructed using the STRING database (v11.0) with *Arabidopsis thaliana* as the reference. Only interactions with a minimum required score (confidence) of 0.700 (high confidence) were considered. Sequence similarity thresholds were set to >30% identity and E-value < 1 × 10^−5^ for orthology assignment.

### 4.5. RT-qPCR Validation

Total RNA was extracted using an RNA extraction kit (SEON, Zhangjiakou, China) and reverse-transcribed into cDNA using a reverse transcription kit (SEON, Zhangjiakou, China). Gene-specific primers were designed and are listed in Supplementary Table S1. The *P. cyrtonema* ubiquitin gene was used as an endogenous reference. RT-qPCR was performed using the TB Green Premix ExTaq kit (Takara, Dalian, China), and relative gene expression levels were calculated using the 2^−ΔΔCt^ method [55]. Transcriptome heatmaps were generated using TBtools -II [56].

### 4.6. Data Analysis and Visualization

Prior to ANOVA, the normality of data distribution was assessed using the Shapiro–Wilk test, and homogeneity of variances was verified using Levene’s test. For each physiological measurement, the exact sample size was *n* = 3 biological replicates. Data were analyzed using one-way ANOVA followed by Tukey’s test (*p* < 0.05). Statistical differences in physiological biochemicalindices, and RT-qPCR data were analyzed using SPSS 22 software. For multiple comparisons following one-way analysis of variance (ANOVA), Tukey’s test was applied, with *p* < 0.05 considered statistically significant. Bar charts were generated and significance labels were added using GraphPad Prism 10. For multiple comparisons following one-way analysis of variance (ANOVA), Tukey’s HSD test was applied for post-hoc analysis, with *p* < 0.05 considered statistically significant. Different letters above the columns in Figure 1 indicate significant differences determined by Tukey’s test.

## 5. Conclusions

In conclusion, this study demonstrates that *P. cyrtonema* responds to continuous drought stress through coordinated physiological adaptations and transcriptomic reprogramming. The identification of a key black co-expression module and candidate hub genes (e.g., *WRKY33* and *RD20*) provides valuable insights into the drought tolerance mechanisms of this medicinal monocot. Together, these findings establish a preliminary physiological and transcriptomic framework for understanding drought responses in *P. cyrtonema*. Although further functional validation is warranted, the identified genetic resources and proposed regulatory networks offer testable hypotheses that lay a solid foundation for future molecular breeding efforts aimed at enhancing stress resilience in this species.

## Data Availability

All data generated or analyzed during this study are included in this article and its Supplementary information files. All materials are available from the corresponding authors upon request.

## Supplementary Materials

The following supporting information can be downloaded at: https://www.mdpi.com/article/10.3390/ijms27219670/s1.
